# Supplementary material for: Enzymatic degradation of RNA causes widespread protein aggregation in cell and tissue lysates
Source: EMBO Rep. 2020 Sep 18;21(10):e49585. doi: 10.15252/embr.201949585 (PMC7534620; doi:10.15252/embr.201949585)
Supplement: Supplementary file 1 — Appendix [file EMBR-21-e49585-s001.pdf]

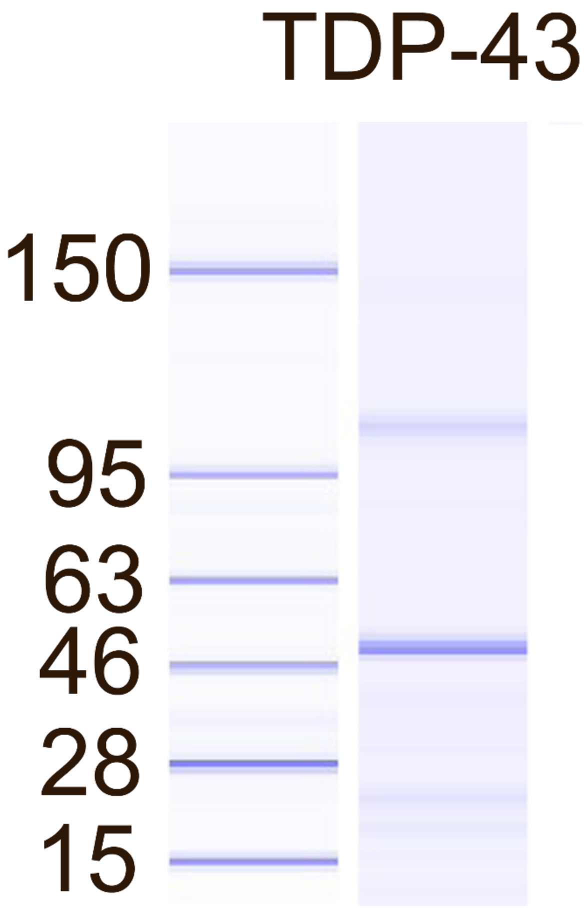

Appendix Figure 1. Recombinant TDP-43.

Recombinant TDP-43 produced in E.coli and analysed on an Agilent Bioanalyzer Protein 230 chip. The band migrating at approximately 46 kDa represents TDP-43. The purity of TDP-43 was judged to be more than 80%.
