# Supplementary material for: Enzymatic degradation of RNA causes widespread protein aggregation in cell and tissue lysates
Source: EMBO Rep. 2020 Sep 18;21(10):e49585. doi: 10.15252/embr.201949585 (PMC7534620; doi:10.15252/embr.201949585)
Supplement: Supplementary file 2 — Expanded View Figures PDF [file EMBR-21-e49585-s002.pdf]

Expanded View Figures

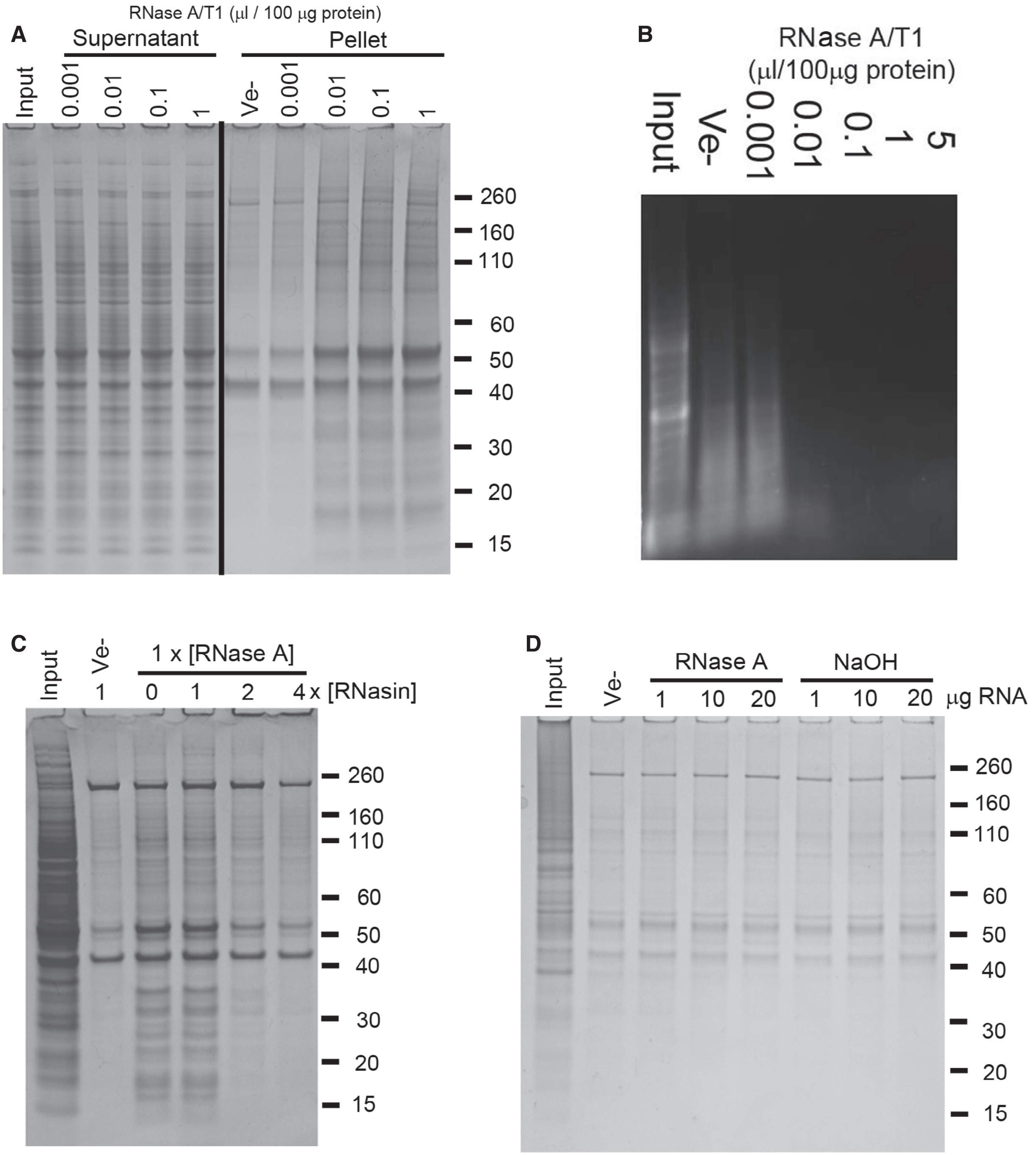

Figure EV1. Protein aggregation caused by enzymatic degradation of RNA, and effect of pre-hydrolysed RNA or RNase inhibition.

A SDS–PAGE analysis of soluble (Supernatant) and aggregated (Pellet) proteins after treatment of mouse brain tissue lysate with increasing amounts of a mixture of RNase A and T1 (A/T1) or vehicle (Ve−).

B Agarose gel electrophoresis analysis of RNA isolated from RNase-treated human neuronal cell lysate.

C, D Insoluble proteins collected by centrifugation after co-treatment of human neuronal cell lysate with RNase A and an RNase A inhibitor (RNasin, C), or following the addition of RNA pre-hydrolysed by RNase A or alkaline hydrolysis (NaOH, D).

 © 2020 The Authors

A

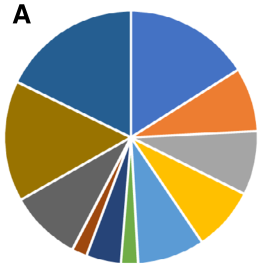

GO Cellular component	No of proteins	%	p value	FDR
protein-containing complex	739	56,1	7.11E-22	1.22E-18
extracellular vesicle	377	28,6	5.20E-19	2.23E-16
extracellular exosome	377	28,5	5.20E-19	2.97E-16
extracellular organelle	376	28,6	4.25E-19	3.64E-16
extracellular space	394	29,9	2.08E-15	7.14E-13
ribosome	101	7,7	4.40E-13	1.26E-10
ribonucleoprotein complex	205	15,6	6.05E-13	1.48E-10
ribosomal subunit	90	6,8	8.56E-13	1.83E-10
extracellular region	421	31,9	4.60E-12	8.77E-10
cytosol	711	53,9	1.59E-11	2.73E-09
membrane	820	62,2	3.86E-11	6.01E-09

B

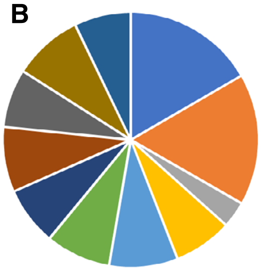

GO molecular function	No of proteins	%	p value	FDR
heterocyclic compound binding	753	57,1	2.17E-21	7.59E-18
organic cyclic compound binding	755	57,3	9.96E-21	1.74E-17
structural molecule activity	151	11,5	3.14E-17	3.66E-14
purine ribonucleotide binding	335	25,4	1.87E-16	1.63E-13
RNA binding	394	29,9	3.38E-16	1.97E-13
nucleotide binding	373	28,3	4.14E-16	2.06E-13
ribonucleotide binding	336	25,5	3.09E-16	2.16E-13
nucleoside phosphate binding	373	28,3	5.58E-16	2.44E-13
purine nucleotide binding	335	25,4	8.77E-16	3.41E-13
purine ribonucleoside triphosphate binding	399	30,3	1.90E-15	6.65E-13
small molecule binding	325	24,7	2.10E-15	6.67E-13

C

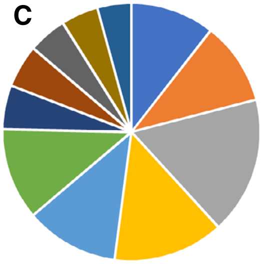

GO biological process	No of proteins	%	p value	FDR
peptide biosynthetic process	151	11,5	1.04E-15	3.32E-12
translation	149	11,3	7.98E-16	3.38E-12
intracellular protein transport	247	18,7	7.89E-16	5.02E-12
cellular amide metabolic process	199	15,1	2.11E-15	5-38E-12
amide biosynthetic process	168	12,7	5.13E-16	6.53E-12
peptide metabolic process	165	12,5	5.29E-15	1.12E-11
viral gene expression	79	6,0	1.24E-14	2.26E-11
translational initiation	77	5,8	3.00E-13	4.78E-10
viral transcription	69	5,2	7.34E-13	1.04E-09
establishment of protein localization to endoplasmic reticulum	67	5,1	2.41E-12	3.07E-09
SRP-dependent cotranslational protein targeting to membrane	61	4,6	3.08E-12	3.56E-09

D

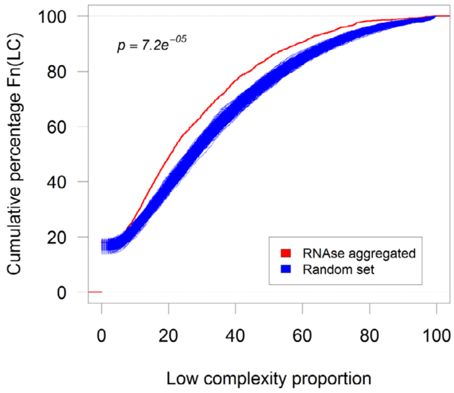

E

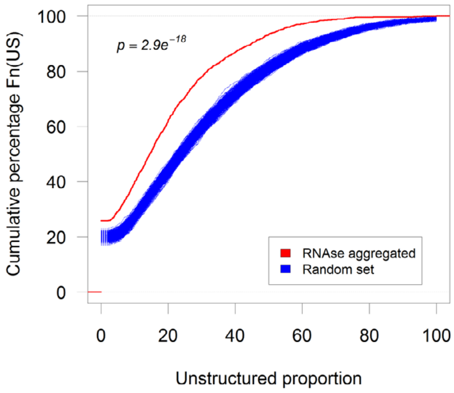

Figure EV2. Computational analysis of proteins aggregated by enzymatic degradation of RNA.

A–C Top ten gene ontology classes by Cellular component (A), Molecular function (B), or Biological process (C).

D, E Cumulative distribution of the proportion of predicted low-complexity regions (D) or unstructured regions (E) in the RNase-aggregated proteins (Red) or random sets of proteins (Blue).

Data information: *P*-values in (D, E) were obtained by the two-sample Kolmogorov–Smirnov test and corrected for multiple testing using Bonferroni correction.

© 2020 The Authors

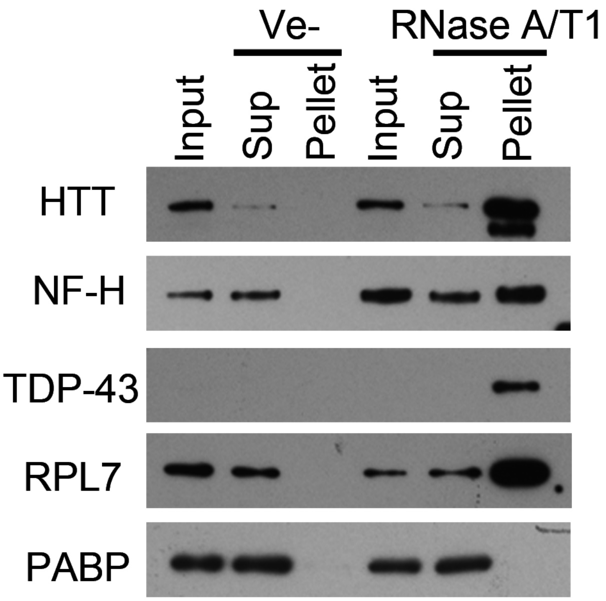

Figure EV3. Protein aggregation caused by enzymatic degradation of RNA in mouse brain tissue lysate.

Western blot analysis of aggregated proteins, collected by centrifugation, after treatment of mouse brain lysate with a mixture of RNase A and RNase T1 (RNase A/T1), or vehicle (Ve−). Input represents the starting material, Sup the supernatant (soluble fraction) and Pellet the aggregated fraction after centrifugation.

 © 2020 The Authors

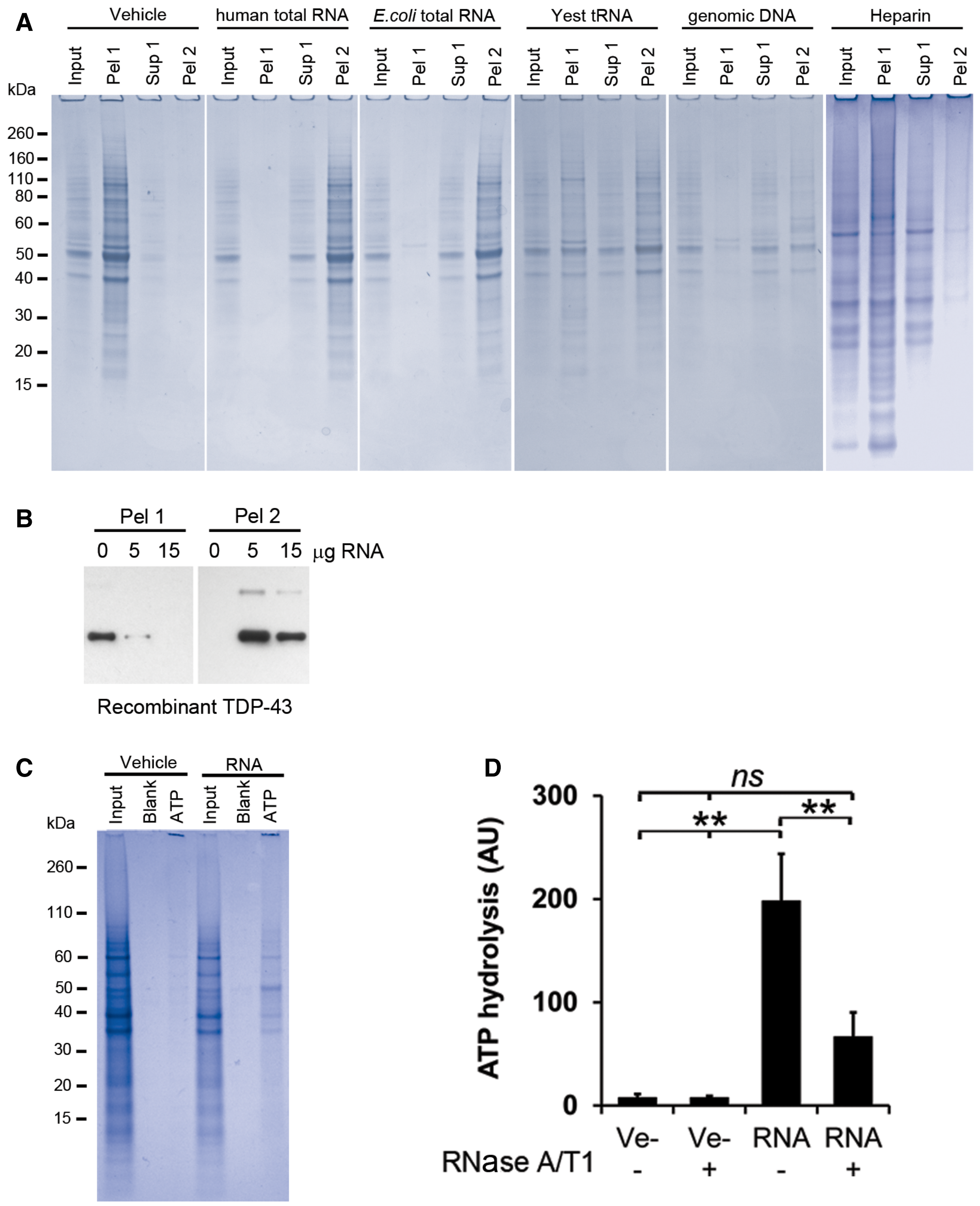

Figure EV4. Protein renaturation: effects on enzymatic activity and effects of various polyanions.

A Coomassie-stained SDS-PAGE gels of RNase-aggregated proteins from human neurons renatured with various polyanions. Input represents 1/10th of each sample, taken before the first centrifugation, and Pel 1 and Sup 1 are the pellet and the supernatant, respectively, recovered after the first centrifugation. Pel 2 represents the pellet obtained after treating the Sup 1 fraction with RNase A/T1 followed by centrifugation. See experimental outline in Fig 3A for a full description.

B Renaturing of recombinant TDP-43 using increasing amounts of total RNA. Pel 1 represents aggregated protein after renaturing. Pel 2 is the aggregated protein after the soluble fraction (Sup 1) has been treated with RNase A/T1.

C Gel electrophoresis analysis of proteins from human neurons renatured with vehicle or total RNA and then captured with ATP-agarose beads (ATP). Input represents 1/10th of each sample taken directly after renaturation, before the first centrifugation, and Blank represents the sample without any ATP-agarose beads present.

D ATP-hydrolysing activity of RNase-aggregated proteins from Jurkat T cells renatured in the presence (RNA) or absence (Ve−) of RNA.

Data information: Data in (C) are expressed in arbitrary units (AU) and represent the mean ± s.d of three independent experiments. $^{**}P < 0.01$ by *post hoc* ANOVA using Bonferroni correction for multiple testing.

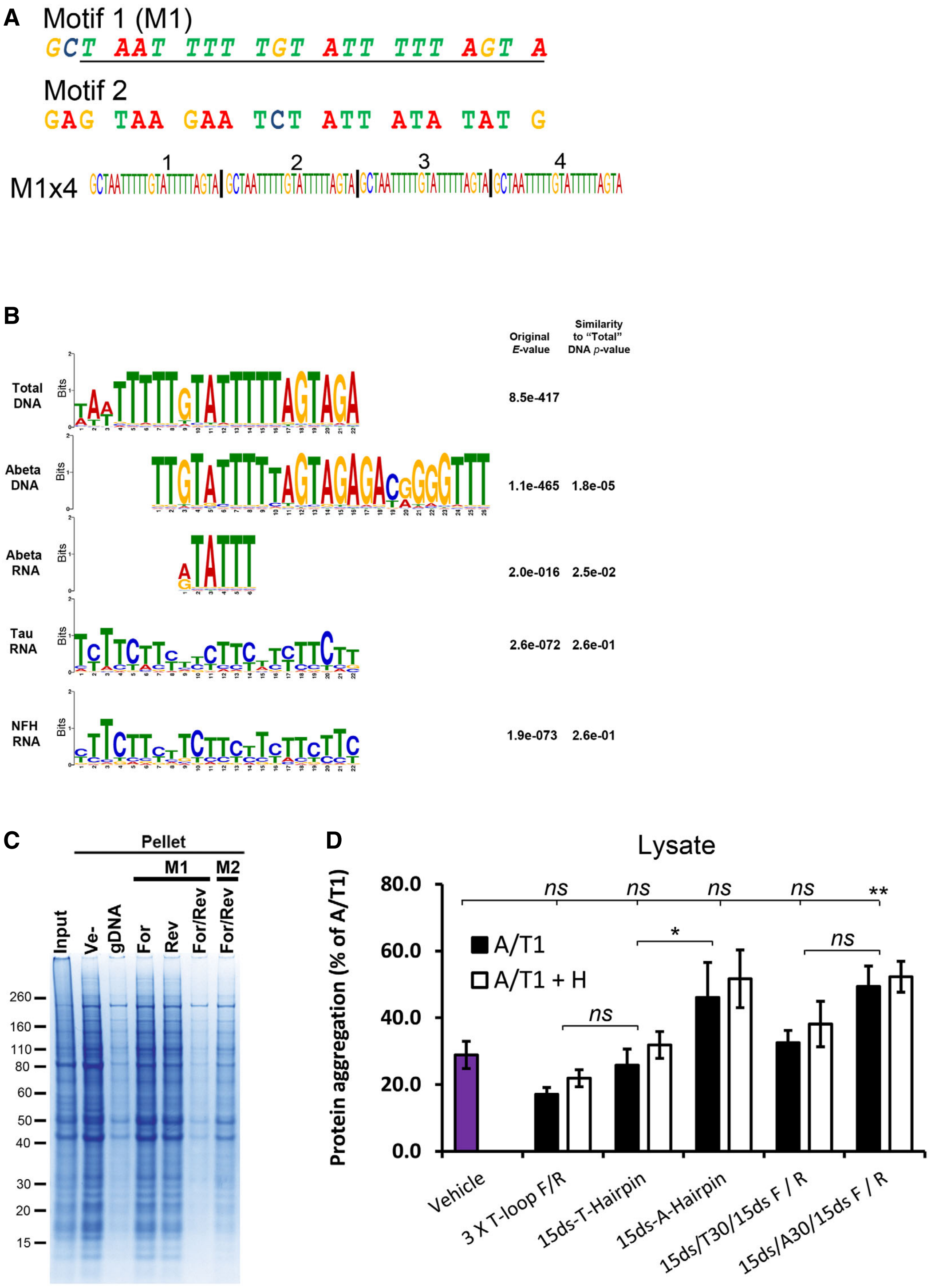

Figure EV5.

 © 2020 The Authors

Figure EV5. Computational and experimental comparison of selected motifs and structural oligonucleotides.

A Sequence of the selected M1 motif and a random control oligo (Motif 2, M2) investigated for their renaturing capacity. Both motifs were assembled into four consecutive repeats of the same motif (e.g. M1x4). The underlined region of M1 represents the computationally identified motif while the two extra 5′ nucleotides (GC) were added for cloning purposes.

B Alignment of motifs identified in the nucleic acids associated with soluble proteins after renaturation. All motifs are compared with the M1 motif identified in the Total DNA samples. The quoted original E-value represents the value obtained in the MEME-Chip analysis and the Similarity *P*-value is the *P*-value given by the TomTom motif comparison software package, compared with the Total DNA motif.

C Coomassie-stained SDS-PAGE gel of aggregated proteins (Pellet) following renaturation with either single-stranded (For or Rev) or complementary (For/Rev) M1x4 or M2x4 DNA oligonucleotides. gDNA represents genomic DNA, and Ve- represents vehicle.

D Amount of protein aggregation observed after treating cell lysate from Jurkat T cells with various oligonucleotides and RNase A/T1 (A/T1) and, if indicated, together with RNase H (A/T1 + H).

Data information: Data in (D) are expressed as a percentage of the aggregation observed in samples treated with RNase A/T1 only and represent the mean ± s.d of three independent experiments. $^{**}P < 0.01$, $^{*}P < 0.05$ by *post hoc* ANOVA using Bonferroni correction for multiple testing.

© 2020 The Authors
