# Supplementary material for: Enzymatic degradation of RNA causes widespread protein aggregation in cell and tissue lysates
Source: EMBO Rep. 2020 Sep 18;21(10):e49585. doi: 10.15252/embr.201949585 (PMC7534620; doi:10.15252/embr.201949585)
Supplement: Supplementary file 3 — Table EV1 [file EMBR-21-e49585-s003.docx]

**Table EV1.**

Oligonucleotides used in this study.

| **Name** | **Sequence** | **Used in Fig:** |
| --- | --- | --- |
| **Abeta_XhoI_F** | CTA GCT CGA GGC CAC CGA TGC AGA ATT CCG ACA TGA | 2b |
| **Abeta_BamHI_R** | CTA GGG ATC CGG ACA ACA CCG CCC ACC ATG A | 2b |
| **TARDBP BspHI_F** | CTA GTC ATG ATG TCT GAA TAT ATT CGG GTA AC | 4b |
| **TARDBP Not I_R** | CTA GGC GGC CGC CAT TCC CCA GCC AGA AG | 4b |
| **M1x4 For** | **GAT CC**G CTA ATT TTT GTA TTT TTA GTA GCT AAT TTT TGT ATT TTT AGT AGC TAA TTT TTG TAT TTT TAG TAG CTA ATT TTT GTA TTT TTA GTA **C** | 5a-d, 6d-e & S6 |
| **M1x3 For** | **GAT CC**G CTA ATT TTT GTA TTT TTA GTA GCT AAT TTT TGT ATT TTT AGT AGC TAA TTT TTG TAT TTT TAG TA**C** | 5c |
| **M1x2 For** | **GAT CC**G CTA ATT TTT GTA TTT TTA GTA GCT AAT TTT TGT ATT TTT AGT A**C** | 5c |
| **M1x1 For** | **GAT CC**G CTA ATT TTT GTA TTT TTA GTA **C** | 5c |
| **M1x4 Rev** | **TCG AG**T ACT AAA AAT ACA AAA ATT AGC TAC TAA AAA TAC AAA AAT TAG CTA CTA AAA ATA CAA AAA TTA GCT ACT AAA AAT ACA AAA ATT AGC **G** | 5a-d, 6d-e & S6 |
| **M1x3 Rev** | **TCG AG**T ACT AAA AAT ACA AAA ATT AGC TAC TAA AAA TAC AAA AAT TAG CTA CTA AAA ATA CAA AAA TTA GC**G** | 5c |
| **M1x2 Rev** | **TCG AG**T ACT AAA AAT ACA AAA ATT AGC TAC TAA AAA TAC AAA AAT TAG C**G** | 5c |
| **M1x1 Rev** | **TCG AG**T ACT AAA AAT ACA AAA ATT AGC **G** | 5c |
| **M2x4 For** | **GAT CC**G AGT AAG AAT CTA TTA TAT ATG GAG TAA GAA TCT ATT ATA TAT GGA GTA AGA ATC TAT TAT ATA TGG AGT AAG AAT CTA TTA TAT ATG **C** |  |
| **M2x4 Rev** | **TCG AG**C ATA TAT AAT AGA TTC TTA CTC CAT ATA TAA TAG ATT CTT ACT CCA TAT ATA ATA GAT TCT TAC TCC ATA TAT AAT AGA TTC TTA CTC **G** | 5a-b, 6d, S6 |
| **ds/T28/ds For** | CTT GCG GCC GCG CGC TTT TTT TTT TTT TTT TTT TTT TTT TTT TGG CCA AGC TTA CCC GCC | 6a, b |
| **ds/T28/ds Rev** | GGC GGG TAA GCT TGG CCT TTT TTT TTT TTT TTT TTT TTT TTT TTT GCG CGC GGC CGC AAG | 6a, b |
| **ds/C28/ds Rev** | CTT GCG GCC GCG CGC CCC CCC CCC CCC CCC CCC CCC CCC CCC CGG CCA AGC TTA CCC GCC | 6a, b |
| **ds/C28/ds For** | GGC GGG TAA GCT TGG CCC CCC CCC CCC CCC CCC CCC CCC CCC CCC GCG CGC GGC CGC AAG | 6a, b |
| **ds/A28/ds Rev** | CTT GCG GCC GCG CGC AAA AAA AAA AAA AAA AAA AAA AAA AAA AGG CCA AGC TTA CCC GCC | 6a, b |
| **ds/A28/ds For** | GGC GGG TAA GCT TGG CCA AAA AAA AAA AAA AAA AAA AAA AAA AAA GCG CGC GGC CGC AAG | 6a, b |
| **ss T50** | TTTTTTTTTTTTTTTTTTTTTTTTTTTTTTTTTTTTTTTTTTTTTTTTTT | 6c |
| **ss A50** | AAAAAAAAAAAAAAAAAAAAAAAAAAAAAAAAAAAAAAAAAAAAAAAAAA | 6c |
| **15ds/T30/15ds For** | GGG CCC GGG CCC GGG TTT TTT TTT TTT TTT TTT TTT TTT TTT TTT GCG CGC GCG CGC GCG | 6c, S6b |
| **15ds/T30/15ds Rev** | CGC GCG CGC GCG CGC TTT TTT TTT TTT TTT TTT TTT TTT TTT TTT CCC GGG CCC GGG CCC | 6c, S6b |
| **15ds/A30/15ds For** | GGG CCC GGG CCC GGG AAA AAA AAA AAA AAA AAA AAA AAA AAA AAA GCG CGC GCG CGC GCG | 6c, S6b |
| **15ds/A30/15ds Rev** | CGC GCG CGC GCG CGC AAA AAA AAA AAA AAA AAA AAA AAA AAA AAA CCC GGG CCC GGG CCC | 6c, S6b |
| **15-Hairpin/T30** | GGG CCC GGG CCC GGG TTT TTT TTT TTT TTT TTT TTT TTT TTT TTT CCC GGG CCC GGG CCC | 6c, S6b |
| **15-Hairpin/A30** | GGG CCC GGG CCC GGG AAA AAA AAA AAA AAA AAA AAA AAA AAA AAA CCC GGG CCC GGG CCC | SbB |
| **15ds/T30 For** | GGG CCC GGG CCC GGG TTT TTT TTT TTT TTT TTT TTT TTT TTT TTT | 6c, S6b |
| **15ds/T30 Rev** | TTT TTT TTT TTT TTT TTT TTT TTT TTT TTT CCC GGG CCC GGG CCC | 6c, S3b |
| **3x9T-loop For** | GGG CCC GGG C TTT TTT TTT T GCA ACT CTT G TTT TTT TTT T C GCG CGC GCG TTT TTT TTT T GGT ACT AGA T | 6c, S6b |
| **3x9T-loop Rev** | A TCT AGT ACC A TTT TTT TTT T CGC GCG CGC G TTT TTT TTT T C AAG AGT TGC TTT TTT TTT T G CCC GGG CCC | 6c, S6b |
| **3x9T-bulge Rev** | A TCT AGT ACC A TT CGC GCG CGC G TT C AAG AGT TGC TT G CCC GGG CCC | 6c |
| **3'-15ds Rev*** | CGC GCG CGC GCG CGC | 6c |
| **5'-15ds Rev*** | CCC GGG CCC GGG CCC | 6c |
| **15T/15ds/15T/15ds For** | TTT TTT TTT TTT TTT GGG CCC GGG CCC GGG TTT TTT TTT TTT TTT GCG CGC GCG CGC GCG | 6c |
| **15T/15ds/15T/15ds Rev** | TTT TTT TTT TTT TTT CGC GCG CGC GCG CGC TTT TTT TTT TTT TTT CCC GGG CCC GGG CCC | 6c |

*Used together with “*15ds/T30/15ds For”* to form 15ds/1xT30/15ds used in Fig 6c. **Bold** sequences represent restriction site overhangs (Bam HI in For and Xho I for Rev), not used in this study. Underlined sequences represent highlight motif repeats.
