# Supplementary material for: Enzymatic degradation of RNA causes widespread protein aggregation in cell and tissue lysates
Source: EMBO Rep. 2020 Sep 18;21(10):e49585. doi: 10.15252/embr.201949585 (PMC7534620; doi:10.15252/embr.201949585)
Supplement: Supplementary file 7 — Movie EV1 [file EMBR-21-e49585-s007.zip › Aarum Ledgend Movie EV1.docx]

**EXPANDED VIEW MOVIE LEGEND**

**Movie EV 1. Effect of RNase-treatment on a clear cell lysate.** Time-lapse movie of Jurkat T-cell lysate treated with RNase A/T1 or vehicle for 30 minutes at room temperature.
